# Functional roles of circular RNAs in lung injury

**DOI:** 10.3389/fphar.2024.1354806

**Published:** 2024-03-27

**Authors:** Fei-Fei Gao, Dian-Qing Chen, Yue-Tong Jiang, Cui-Fei Han, Bi-Yun Lin, Zhan Yang, Juan-Hua Quan, Ying-Huan Xiong, Xin-Tian Chen

**Affiliations:** ^1^ Stem Cell Research and Cellular Therapy Center, Affiliated Hospital of Guangdong Medical University, Zhanjiang, Guangdong, China; ^2^ Department of Hand and Foot Surgery, Armed Police Corps Hospital of Hebei, Shijiazhuang, Hebei, China; ^3^ Guangdong Medical University, Zhanjiang, Guangdong, China; ^4^ Biotissue Repository, Affiliated Hospital of Guangdong Medical University, Zhanjiang, Guangdong, China; ^5^ Laboratory of Gastroenterology, Affiliated Hospital of Guangdong Medical University, Zhanjiang, Guangdong, China

**Keywords:** circRNA, lung injury, inflammation, macrophage, fibrosis, smoking

## Abstract

Lung injury leads to respiratory dysfunction, low quality of life, and even life-threatening conditions. Circular RNAs (circRNAs) are endogenous RNAs produced by selective RNA splicing. Studies have reported their involvement in the progression of lung injury. Understanding the roles of circRNAs in lung injury may aid in elucidating the underlying mechanisms and provide new therapeutic targets. Thus, in this review, we aimed to summarize and discuss the characteristics and biological functions of circRNAs, and their roles in lung injury from existing research, to provide a theoretical basis for the use of circRNAs as a diagnostic and therapeutic target for lung injury.

## 1 Introduction

Lung injury, which can be caused by sepsis, pneumonia, trauma, aspiration pneumonia, and even some treatments, leads to respiratory dysfunction, and seriously affects the quality of life (Matthay et al., 2019). Acute lung injury (ALI) has a high morbidity and mortality of approximately 30%. When the lung tissue fails to fully repair, the lung inflammatory responses may ultimately lead to chronic obstructive pulmonary disease, which is the fourth leading reason of death globally (Tsushima et al., 2009; Pelgrim et al., 2019). Even there are some studies focused on human embryonic stem cells (Wu et al., 2020), utilizing lung spheroid cell-secretome (LSC-Sec) and exosomes (LSC-Exo) for lung injury and fibrosis treatments (Dinh et al., 2020), the challenges of consistency, safety, and clinical applicability of those therapies are not be ignored. Therefore, fully elucidating the underlying development mechanism of lung injury is expected to fundamentally improve the treatment.

Circular RNAs (circRNAs) exist widely. Several specialized computational tools and databases based on different identification strategies have been combined with next-generation sequencing and bioinformatic analysis to identify and analyze circRNAs(Chen et al., 2021). circRNAs are reported to be not only involved in cardiovascular biology (Aufiero et al., 2019), brain injury (Zhu et al., 2020), kidney-related diseases (Chen et al., 2021), but also tumor progression (Shang et al., 2019). However, the knowledge regarding why circRNAs exist in various diseases remains limited, and potential roles in lung injury progression are unclear.

**FIGURE 1:**
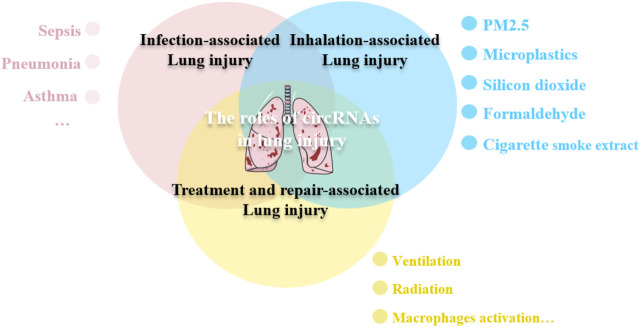
circRNAs participate in the lung injury. Lung injury can be caused by various etiologies, including infection, toxic substances inhalation, even some treatments maybe the contributing injury factors. circRNAs are involved in the pathological process of lung injury through different mechanisms.

To this end, we aimed to review the characteristics of circRNAs and discuss their potential roles in lung injury caused due to multiple factors. The finding of this review may aid in underscoring the potential of circRNAs to be used as a target for the diagnosis and treatment of lung injuries (Figure 1).

## 2 Formation and characteristics of circRNAs

CircRNAs range from <100 nucleotides to multiple kilobases (Lasda and Parker, 2014; Aufiero et al., 2019). CircRNAs including exonic circRNAs (ecircRNAs), circular intron circRNAs (ciRNAs), and exon- and intron-derived or retained intron circRNAs (EIciRNA) (Han et al., 2017), are formed by intron pairing, RNA-binding protein, and lariat-driven circularization mechanisms (Aufiero et al., 2019).

These different splicing mechanisms confer consistent characteristics on circRNAs. They are highly conserved in different tissues and conditions; the covalently closed loop structures endow them with RNase resistance, thereby providing them with the properties of a biomarker (Li et al., 2015). Moreover, the specific location and expression of circRNAs lead to different biological functions. The majority of circRNAs are ecircRNAs, which are located in the cytoplasm and can interact with target miRNAs, thereby acting as miRNA sponges or reservoirs. The miRNA sponges cause an increase in the expression of target mRNAs, whereas the miRNA reservoir decreases the target mRNA expression (Memczak et al., 2013; Qu et al., 2015).

## 3 circRNAs in lung development

circRNAs have been reported to be involved in the development of the human brain, kidney, and liver (Xu et al., 2017). By analyzing the database analysis available on the circBase database, 9,698 circRNA candidates have been detected in fetal lung tissues, which is eight times more than those found in adult lung tissues. RNA sequencing analysis in humans has further verified that the expression of 1,701 circRNAs in fetal lung samples is higher than that in the corresponding adult lung. 452 unique circRNAs are enriched in the lung than in the other organs, suggesting that circRNAs may play crucial roles in human lung development (Xu et al., 2017; Tong et al., 2023).

Bronchopulmonary dysplasia (BPD) is the most common complication associated with extremely preterm infants and its prevalence has been increasing worldwide (Thébaud et al., 2019). circABCC4 promotes BPD progression by facilitating PLA2G6 expression by sequestering miR-663a (Chen et al., 2020). PLA2G6, which belongs to the phospholipase A2 family that is involved in signal transduction and phospholipid homeostasis (Deng et al., 2023), further aggravates lung inflammation by promoting the production of arachidonic acid metabolites (Bellido-Reyes et al., 2006). circABPD1 was also found highly expressed in preterm colostrum milk exosomes, it can alleviate lung injury by targeting the miR-330–3p/HIF1α axis (Li et al., 2023). Three upregulated circRNAs (hsa_circ_0005389, hsa_circ_0000367, and hsa_circ_0059571) and two downregulated circRNAs (hsa_circ_0058495, hsa_circ_0006608) were found in neonatal acute respiratory distress syndrome (NARDS) through high-throughput sequencing in ten clinical blood samples of newborns (Zhou et al., 2021). These findings provide a new therapeutic direction to use circRNAs as molecular markers for early diagnosis of lung injury; nevertheless, existing studies focus only on the changes in circRNA expression levels, based on human or animal models, dynamic observation of circRNA with neonates at different stages as research objects may be solid evidence in clarifying the production and function of circRNAs in lung development.

## 4 circRNAs in infection-associated lung injury

**FIGURE 2:**
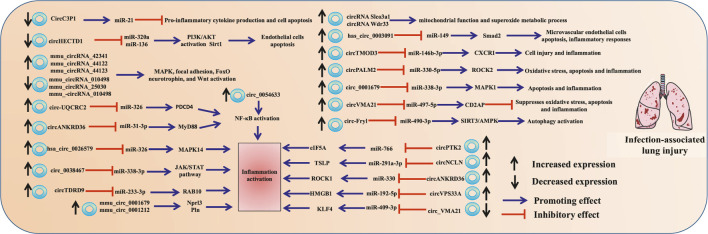
circRNAs participate in infection-associated lung injury. The schematic diagram depicts the known role of circRNAs in infection-associated lung injury progression.

Sepsis is a systemic inflammatory response syndrome that is triggered by infection with pathogenic bacteria, viruses, or fungi; it is also the major cause of ALI (Wang et al., 2018). Lipopolysaccharide (LPS) is a vital medium for sepsis. The role of circRNAs in infection-associated lung injury has been validated primarily by using clinical sample combined with multiple models of LPS-induced lung injury *in vivo* and *in vitro* (As shown in Figure 2).

Some circRNAs are found in samples of patients with ALI. Compared to those in healthy controls, 35 circRNAs were upregulated and 9 were downregulated in patients with sepsis, hsa_circ_0003091 (mmu_circ_0015268) were found to be significantly elevated both in ALI patient and mice. Mechanically, hsa_circ_0003091 sponged miR-149 to upregulate the expression of Smad2, thereby contributing to pulmonary injury, cell apoptosis, and inflammatory responses (Shen et al., 2022). Rho-associated coiled-coil-containing protein kinase I (ROCK1), a member of the serine/threonine protein kinase family, primarily exists in the lung tissues and exhibits facilitating effect on inflammation in ALI (Ishizaki, 2003; Meng et al., 2019). circANKRD36 expression was significantly elevated in the serum of patients with sepsis-induced ALI. circANKRD36 serves as a sponge for miR-330, leading to the increase of ROCK1 expression and, aggravating inflammation of LPS-stimulated RAW264.7 cells (Lin et al., 2021). Programmed cell death 4 (PDCD4), a well-known tumor suppressive protein has been demonstrated as a novel modulator in inflammation response by activating several inflammatory signaling, including the NF-κB pathway (Su et al., 2015). circ-UQCRC2 is upregulated in the serum of patients with pneumonia and LPS-treated MRC-5 cells. circ-UQCRC2 directly target miR-326 to upregulate PDCD4 expression for the activation of NF-κB pathway (Zhou et al., 2021).

Mitogen-activated protein kinase 14 (MAPK14) is ubiquitously expressed in various cell types and, exhibits a vital role in response to inflammation (Zhang et al., 2021). hsa_circ_0026579 (circESPL1) expression is significantly upregulated in patients with pneumonia and acts as a sponge of miR-326 for MAPK14 activation during LPS-induced lung cell injury (Liang et al., 2022).

The roles of circRNA in lung injury have been validated both *in vivo* and *in vitro*. 20 circRNAs were found to be upregulated and 18 were downregulated in ALI mice induced by cecal ligation and puncture. These circRNAs were found to be closely associated with the inflammatory response (e.g., the TGF-β, MAPK, Fc gamma R-mediated phagocytic, TNF, and chemokine signaling pathways) using bioinformatics analyses (Yuan et al., 2020; Teng et al., 2021). circPTK2 was upregulated in cecal ligation and puncture-based mouse and LPS-based alveolar type II cell (RLE-6TN), and reasonable for the ATP efflux, pyroptosis, and inflammation through upregulating eIF5A expression by competitively adsorbing miR-766 (Ding et al., 2023). Similarly, upregulated circTDRD9 acted as miR-223-3p sponge to increase RAB10 expression, also promoting LPS-induced lung injuries (Zhang et al., 2023).

CircRNAs could play a protective role against lung injury. Reportedly, 21 upregulated and 55 downregulated circRNAs are involved in the progression of LPS-induced autophagy in human bronchial epithelial cell 16HBE(Liu et al., 2021). Furthermore adipose-derived stem cell exosomes have high levels of the circular RNA (circ)-Fryl, which plays a protective role against sepsis-induced mouse lung injury by decreasing apoptosis and inflammatory factor expression. Mechanistically, miR-490-3p and SIRT3 are downstream targets of circ-Fryl. circ-Fryl overexpression promotes autophagy by inducing SIRT3/AMPK signaling and sponging miR-490-3p (Shen et al., 2022).

Moreover, circC3P1 is downregulated in ALI mice induced by sepsis; it attenuates pro-inflammatory cytokine production and cell apoptosis through the modulation of miR-21 (Jiang et al., 2020). Elevated circVMA21 levels suppress oxidative stress, apoptosis, and inflammation via mediating the miR-497-5p/CD2AP axis to mitigate ALI in sepsis rats (Ke et al., 2022). circ_0038467 knockdown alleviates LPS-induced inflammatory injury in 16HBE cells by sponging miR-338-3p and inhibiting the activation of JAK/STAT3 pathway (Liu et al., 2020). Similarly, circHECTD1 is downregulated in LPS-induced human and mouse AECs [HBE and murine lung epithelial-12 (MLE-12)]; it inhibits the apoptosis of AECs through the miR-320a/PIK3CA and miR-136/Sirt1 pathways (Li et al., 2022).

Phospholamban (Pln), cadherin-2 (Cdh2) and Nprl3 are found to participate in the pathogenesis of sepsis and promote inflammation (Black et al., 2018; Weng et al., 2019; Zhuang et al., 2020). mmu_circ_0001679 is reported to regulate the expression of Nprl3, and mmu_circ_0001212 similarly regulates Pln, Cdh2 and Nprl3 expression, which were all increased in the sepsis mice (Zou et al., 2020). ROCK2 aggravates sepsis-caused ALI through association with miR-424 and transendothelial migration of polymorphonuclear leukocytes (Li et al., 2010; Chen et al., 2020). CircPALM2 is increased, and involved in LPS-caused MLE-12 cell damage by targeting miR-330-5p, thereby leading to ROCK2 activation (Ren et al., 2022).

circRNAs can aggravate lung injury by maintaining the activation of the NF-κB, MAPK, and WNT pathways. Alveolar epithelial cell-produced thymic stromal lymphopoietin (TSLP) has been shown to worsen ALI by triggering airway inflammation. miR-291a-3p can directly bind to the 3′-UTR of TSLP and suppress TSLP expression. circNCLN has been identified to act as a sponge to antagonize miR-291a-3p and thereby maintain the expression of TSLP (Cao et al., 2022). circ_0054633 is over-expressed in LPS-induced rats and murine pulmonary microvascular endothelial cells, through activating the NF-κB pathways (Yang et al., 2021). Similarly, circANKRD36 is upregulated in LPS-induced MRC-5 cells, and associated with cell injury through regulating miR-31/MyD88-mediated activation of the NF-κB pathway (Guo et al., 2020).

Activation of the MAPK and Wnt pathways are responsible for neutrophil infiltration and pro-inflammatory cytokine production (Cheng et al., 2018). Circ_0001679 is upregulated in LPS-induced MLE-12 cells, and maintains a high expression of MAPK1 by suppressing miR-338-3p, leading to the increased apoptosis (Lu et al., 2022). It is found that mmu_circRNA_42341, mmu_circRNA_44122, and mmu_circRNA_44123 were substantially upregulated, whereas mmu_circRNA_010498, mmu_circRNA_25030, and mmu_circRNA_010498 were significantly downregulated through microchip analysis. These differentially expressed circRNAs were chiefly involved in the MAPK and Wnt signaling pathways (Li et al., 2019).

C–X–C motif chemokine receptor 1 (CXCR1) is necessary for the activation of inflammatory mediators, CXCR1 antagonism has been proposed as a protective strategy against bacterial pneumonia (Wei et al., 2013; Ha et al., 2017). LPS upregulates circTMOD3 expression in normal lung fibroblast (WI-38) cells, and circTMOD3 functions as a competing endogenous RNA for miR-146b-3p to induce CXCR1 expression (Ma et al., 2021). Similarly, Kruppel-like transcription factor 4 (KLF4) is an inflammatory palliative in sepsis (Li et al., 2018). circ_VMA21 was downregulated in pneumonia samples and LPS-treated WI-38 cells, and circ_VMA21 could sponge miR-409-3p to induce the expression of KLF4 (Wang et al., 2021).

In addition to bacterial inflammation, lung injury can be caused by other pathogens. circRNAs Slco3a1 and Wdr33 were aberrantly expressed in the plasma of influenza A virus-induced ALI patients. Biological process analysis revealed that both circRNAs might be involved in the mitochondrial function and superoxide metabolic process (Wang et al., 2021). Moreover, house dust mite is the major allergen contributor to asthma, circRNAs vacuolar protein sorting 33A (circVPS33A, circ_0000455) was highly expressed in a murine asthma model and *Dermatophagoides pteronyssinus* peptidase 1-treated BEAS-2B cells. circVPS33A targeted miR-192-5p to upregulate the expression of high-mobility group box 1 (HMGB1), a strong pro-inflammatory mediator in the pathogenesis of asthma, leading to lung injury (Imbalzano et al., 2017; Su et al., 2021).

## 5 circRNAs in inhalation lung injury

Inhalation exposure to toxic substances, such as PM2.5, polystyrene microplastics (PS-MPs), phagocytosis of silicon dioxide (SiO_2_), formaldehyde (FA), and cigarette smoke extract (CSE) could compromise respiratory epithelial barrier integrity and induce inflammation and lung injury. Multiple studies have defined circRNAs as potential disease modifier in lung injury caused by multiple environmental factors (Table.1).

**TABLE 1:** circRNAs are involved in inhalation-induced lung injury and lung treatment and repair. Multiple circRNAs are involved in lung injury and repair.

Model	circRNAs	Expression	Target miRNA	miRNA Targeted genes	Function
The lung and BALF of mice exposed to PM_2.5_	circBbs9 (Li et al., 2020)	Increased	miR-30e-5p	NLRP3	Inflammation aggravation
The blood, BALF, and lung tissues of rat exposed to polystyrene microplastics	circRNA014924 circRNA006603	Increased	Undefined	Undefined	Respiratory epithelial barrier integrity compromise, lung injury, inflammation
	circ003982 (Fan et al., 2022)	Decreased			
The lung tissues and bronchoalveolar lavage fluid of rats exposed to microplastics, mouse alveolar epithelial cells	circ_kif26b (Luo et al., 2023)	Increased	miR-346-3p	p21	Inflammation activation
The lung tissues of mice exposed to SiO_2_, and primary alveolar macrophages from patients with silicosis	circHECTD1 (Fang et al., 2018)	Increased	Undefined	HECTD1	Endothelial–mesenchymal transition promotion
The lung tissues of silicosis mice, macrophages from patients with silicosis and RAW264.7 and L929 exposed to SiO_2_	circHECTD1 (Zhou et al., 2018)	Decreased	Undefined	HECTD1 ZC3H12A	Fibroblast activation, silicosis progression
The peripheral serum of silicosis patients, silicosis mouse model and silica-stimulated macrophages and fibroblasts	circRNA11:120406118|12040782 (Zhang et al., 2023)	Increased	miR-30b-5p	NLRP3	Aggravating macrophages pyroptosis
The lung tissues and BALF of rat exposed to formaldehyde	Circular RNA-CDR1 (Liu et al., 2021)	Increased	rno-miR-7b	ATG7	Autophagosomes formation
The lung tissues of rat and RTE cells exposed to formaldehyde	rno_circRNA_008646 (Plasschaert et al., 2018)	Increased	rno-miR-224	FOXI1	Airway cystic fibrosis
The lung tissues of SD rats exposed to formaldehyde, and primary alveolar cells from male adult SD rats	circRNA_006061 (Ge et al., 2023)	Increased	rnomiR-128-3p	p38/ATF3	Aggravated lung injury
The lung tissues, BALF from CSE-induced mice, and CSE-treated murine alveolar epithelial cells	circFOXO3 (Zhou et al., 2021)	Increased	miR-214-3p	IKK-β	NF-κB activation
The lung tissues of smokers and CSE-induced HPMECs	circANKRD11 (Wang et al., 2021) circ-OSBPL2 (Zheng et al., 2021)	Increased	miR-145-5p miR-193a-5p	BRD4	Oxidative stress and inflammation promotion
The lung tissues of mice exposed to CSE, and CSE-induced bronchial epithelial cells, and embryonic lung fibroblast	circRNA_0026344 (Bai et al., 2021)	Decreased	miR-21	Smad7, TGFβ1/Smad3 activation	Fibroblast differentiation and ECM deposition
Lung tissue from COPD smokers, smokers, and matched non-smokers and alveolar epithelial cells exposed to CSE	circRNA_0026344 (Bai et al., 2021)	Decreased	miR-21	PTEN pathway inhibition	ERK pathway activation, and increased autophagy, apoptosis
Cd-induced mouse lung tissue and blood, bronchial epithelial cell lines	circCIMT(Li et al., 2023)	Decreased	Undefined	APEX1	DNA damage
Lung tissue from NSCLCs patients, smokers, and matched non-smokers, alveolar epithelial cells, and CSE-induced macrophages	circEML4 (Cheng et al., 2023)	Increased	Undefined	ALKBH5	JAK-STAT pathway activation
Lung tissue from COPD patients, lung cancer patients, COPD patients with lung cancer, alveolar epithelial cells	CircTMEM30A (Shen et al., 2023)	Increased	miR-130a	TNFα	COPD and lung cancer aggravation
The lung tissues of smokers and CSE-stimulated bronchial epithelial cells	Circ_0006892 (Zhang et al., 2022)	Decreased	miR-24	PHLPP2	Alleviating bronchial epithelial cell apoptosis
The lung tissues of high-tidal volume ventilation-induced lung injury mice	novel_circ_0000899 novel_circ_0014815	Increased	Undefined	Undefined	Regulation of metabolic processes, protein phosphorylation, and chromatin organization; Ras, rap1, PI3K−Akt signaling pathways
	novel_circ_0015069 (Chen et al., 2022)	Decreased	Undefined	Undefined	
The PBMC and monocytes from patients with sepsis; blood, BALF and lung tissues of mice; MH-S, SV40 and Raw264.7 cells	circN4bp1 (Zhao et al., 2021)	Increased	miR-138-5p	EZH2	M1 polarization
The lung tissues of septic-induced mice and MLE-12 cells	circ_0001679 (Zhu et al., 2022)	Increased	miR-338-3p	DUSP16	Apoptosis and proinflammatory
Peripheral blood mononuclear cells of children with asthma, healthy controls, and CRE-induced mouse	circS100A11 (Liang et al., 2023)	Increased	Undefined	CAPRIN1	S100A11 translation, promoting STAT6 expression, M2 macrophage activation
The lung tissues of mice exposed to SiO_2_, and SiO_2_-induced bronchial epithelial cells	circPWWP2A (Hou et al., 2023)	Increased	miR-223–3p	NLRP3	Pulmonary fibrosis aggravation
The lung tissues of mouse exposed to SiO_2_	hsa_circ_0006916 (Wu et al., 2023)	Increased	Undefined	TGF-β1	M2 macrophage activation
The blood from patient with traumatic lung injury	hsa_circRNA_102,927 hsa_circRNA_100,562	Decreased	Undefined	Undefined	mTOR
	hsa_circRNA_101,523 (Jiang et al., 2020)	Increased			Ras, Relaxin pathways activation
The lung tissues from thoracic irradiation- mice	circRNA4146, circRNA4584, circRNA5229, circRNA544, circRNA1092, circRNA3340 (Li et al., 2021)	Increased	Undefined	Undefined	Th1 and Th2 differentiation pathways

PM2.5 inhalation upregulates the expression of circBbs9, which binds to miR-30e-5p for the activation of NLRP3, aggravating lung inflammation (Li et al., 2020). circRNA 014924 and circRNA 006603 were upregulated and circ003982 was downregulated in the rat lung tissues on PS-MP exposure (Fan et al., 2022). What’s more, PS-MPs inhalation increased circ_kif26b levels in alveolar epithelial cells, which upregulated the expression p21 by binding to miR-346-3p. The increased p21 expression activated SASP and increased the secretion of inflammatory factors IL-6 and IL-8, promoting alveolar epithelial cell senescence and participating in inflammatory lung injury (Luo et al., 2023)

Similarly, SiO_2_ exposure is the biggest promoter of silicosis. SiO_2_ exposure downregulates circHECTD1 levels and increased HECTD1 protein expression. The increased HECTD1 protein expression is associated with macrophage activation and contributing to the progression of silicosis (Fang et al., 2018; Zhou et al., 2018). It is found that circRNA11:120406118|12040782 was increased in the peripheral serum of silicosis patients, which facilitated the progress of silicosis by aggravating NLRP3-mediated macrophages pyroptosis through sponging miR-30b-5p (Zhang et al., 2023).

Formaldehyde, a prevailing air pollutant, has seriously threatened public health in recent years (Zhao et al., 2021). Long-term formaldehyde inhalation upregulates the expression of circRNA-CDR1 in rat lung tissues in a dose-dependent manner. Mechanistically, circRNA CDR1 suppresses rno-miR-7b to elevate ATG7 expression, which is necessary for the formation of autophagosomes, consequently resulting lung injury (Tanida et al., 2012; Liu et al., 2021). Similarly, rno_circRNA_008646 and circRNA_006061 were also significantly high in rat lung tissues when exposed to formaldehyde (Yang et al., 2022). rno_circRNA_008646 sponges rno-miR-224 to upregulate the expression of forkhead box I1 (FOXI1) (Plasschaert et al., 2018), and circRNA_006061 activated p38/ATF3 pathway expression via sponging the rnomiR-128-3p (Ge et al., 2023), contributing to airway cystic fibrosis.

To date, evidence indicates that smoking, including e-cigarettes, also can induce lung inflammation and injury. The roles of circRNAs in CSE-induced lung injury cannot be overlooked. circFOXO3 is significantly upregulated in cigarette smoke-exposed mice lungs and CSE-treated murine alveolar epithelial cells. circFOXO3 sponge miR-214-3p to the upregulate IKK-β mRNA, thereby resulting in NF-κB signaling activation (Zhou et al., 2021). Bromo-domain-containing 4(BRD4) participates in promoting inflammation and oxidative stress (Song et al., 2020). circANKRD11 and circ-OSBPL2 are highly expressed in the lung tissues of smokers and CSE-induced human pulmonary microvascular endothelial and bronchial epithelioid cells (Wang et al., 2021). circANKRD11 can sponge miR-145-5p to upregulate the expression of BRD4, and circ-OSBPL2 serve as a sponge for miR-193a-5p, which also upregulates BRD4 in HBECs(Zheng et al., 2021). Meanwhile, circRNA_0026344 is downregulated in CSE-induced bronchial epithelial cells, with increasing levels of miR-21. The elevated miR-21 can be transported to bronchial fibroblasts through exosomes, leading to the inhibition of Smad7 expression and activation of the TGFβ1/Smad3 pathway, thereby contributing to bronchial fibroblast differentiation and ECM deposition (Bai et al., 2021). In alveolar epithelial cells, CS decreases circRNA_0026344 levels, which sponges miR-21 to inhibit the PTEN, leading to the activation of ERK pathway and increased autophagy and apoptosis, contributing to emphysema (Bai et al., 2021).

The persistent lung inflammation induced by the inhalation of environmental pollutants causes chronic morbidity, as well as leads to sudden and fatal lung dysfunction. It is also reported that CSE-induced circRNAs are associated with tumor progression. Exposure of human lung tissue to Cadmium (Cd) is mainly through the inhalation of cigarette smoke and airborne particulate. Lower circCIMT expression was associated with DNA damage in the mouse lung tissue and blood after Cd exposure, which contributing to the acquisition of tumor characteristics of lung epithelial cells (Li et al., 2023). What’s more, smoking-induced M2 macrophages via circEML4 in extracellular vesicles promote the non small cell lung cancer progression through ALKBH5-regulated m6A modification of SOCS2, which leading to the activation of JAK-STAT pathway (Cheng et al., 2023). Similarly, CircTMEM30A was highly expressed in COPD patients with lung cancer, and it regulated the expression of TNFα through miR-130a, thereby promoting the progression of COPD and lung cancer (Shen et al., 2023). Whereas, circRNAs have also been reported to play protective roles in CSE-induced lung injury. PH domain and leucinerich repeat protein phosphatase 2 (PHLPP2) inhibit inflammation in the progression of lung cancer and injury (Gu et al., 2018; Yan et al., 2018). circ_0006892 is downregulated in lung tissues of smokers and CSE-stimulated bronchial epithelial cells. It can promote PHLPP2 expression via regulating miR-24 and alleviating CSE-induced apoptosis and inflammatory response (Zhang et al., 2022).

## 6 circRNAs in lung treatment and repair

Pulmonary dysfunction caused by lung injury triggers a self-repair process and may partly require the auxiliary treatment of mechanical ventilation. Improper use of a ventilator can worsen lung injury. Numerous significant circRNAs likely participate in the pathological process (Table.1).

Compared to those in the control group, 171 circRNAs were significantly upregulated and 114 were significantly downregulated in the lung tissues of high-tidal volume ventilation-induced mice. novel_circ_0000899 and novel_circ_0014815 were identified to be the most upregulated circRNAs, whereas novel_circ_0015069 was the most downregulated circRNA. These circRNAs were found to be involved in metabolic processes, and in the pathway of Ras, Rap1, and PI3K/Akt (Chen et al., 2022).

Macrophages can be activated and polarized in response to lung injury. The classically activated pro-inflammatory macrophage (M1) and alternatively activated anti-inflammatory macrophage (M2) have been extensively investigated in lung injury, repair, and fibrosis (Sica and Mantovani, 2012; Cheng et al., 2021). The circRNA expression patterns in macrophage activation in lung injury were analyzed by many studies.

11 and 126 circRNAs were found to be significantly upregulated and downregulated, respectively, in pulmonary macrophage polarization. Further biological analysis revealed that the upregulated circRNAs were involved in mitochondrion distribution regulation and Notch binding, whereas the downregulated ones were primarily mainly involved in histone H3K27 methylation (Bao et al., 2019).

EZH2, a histone methyltransferase, is involved in sepsis-induced inflammation and lung injury through modulating macrophage M1 polarization (Zhang et al., 2019). circN4bp1was overexpressed in PBMC and monocytes, and was correlated with a poor prognosis in sepsis induced ALI patients. circN4bp1 can sponge miR-138-5p for the expression of EZH2 (Zhao et al., 2021). Similarly, dual-specificity phosphatases 16 (DUSP16) could be inducible in macrophages, and negatively regulate the JNK pathway to attenuate metabolic stress-triggered hepatic steatosis (Zhang et al., 2015; Wu et al., 2020). circ_0001679 was overexpressed in sepsis-induced ALI mice and MLE-12 cells. It bound to mmu-miR-338-3p and miR-338-3p targeted DUSP16 3′-UTR to reduce DUSP16 expression and aggravate injury (Zhu et al., 2022). circS100A11 was dominantly expressed in monocytes and significantly upregulated in children with asthma, reasonably for the M2 macrophage activation. Mechanistically, circS100A11 promoted S100A11 translation, which liberated SP3 from nucleolin and increased STAT6 expression (Liang et al., 2023). Similarily, circPWWP2A could adsorb miR-223–3p to regulate NLRP3 after silica stimulation in pulmonary fibrosis (Hou et al., 2023), and hsa_circ_0006916 was upregulated in pulmonary fibrosis, associated with the high expression level of M2 molecule TGF-β1, playing an important role in the activation of M1-M2 polarization (Wu et al., 2023).

## 7 Conclusion and perspectives

In this review, we summarized the origin and functions of circRNAs, and discussed their roles in lung development and injury caused by different etiologies. However, their role in lung injury remains mostly unelucidated, and the functions of most circRNAs are still not fully analyzed and require further exploration.

First, various factors can cause lung injury. Hemorrhagic shock and thoracic trauma can lead to lethal lung injuries. Reportedly, 13 circRNAs were significantly upregulated and 16 were downregulated in hemorrhagic shock-induced ALI rat lung tissues; these circRNAs might participate in DNA damage recognition and repair (Wang et al., 2021). Furthermore, downregulated hsa_circRNA_102,927 and hsa_circRNA_100,562, and upregulated hsa_circRNA_101,523 were identified in the plasma samples of patients with traumatic lung injury (Jiang et al., 2020). Radiation-induced lung injury (RILI) is a key threat to patients who undergo thoracic radiotherapy, in the thoracic irradiation-induced RILI mice, 10 circRNAs were downregulated and 17 were upregulated (including circRNA4146, circRNA4584, circRNA5229, circRNA544, circRNA1092, and circRNA3340), which are reported related to the Th1 and Th2 differentiation pathways (Li et al., 2021). Those results suggest that circRNAs are involved in the process of lung injury caused by various etiologies but are limited to undefined specific pathology. Current studies are mainly focused on the relationship between circRNA and a certain signaling pathway in a lung injury-related model, and changes in signaling pathways further regulate inflammatory. The crosstalk of various pathways of inflammatory, possible role of circRNA in the regulation of those pathways, and whether some specific circRNAs are involved in all the processes of lung injury caused by multiple etiologies still need to be explored.

Second, circRNAs are universal and stable, and may serve as novel biomarkers. The downregulated hsa_circRNA_042882 and upregulated hsa_circRNA_104034 in bronchoalveolar lavage fluid were regarded as promising diagnostic biomarkers for patients with ARDS caused by severe pneumonia (Sun et al., 2023). Blood samples are mainly collected from patients for the current study of lung injury. Whether circRNAs exist in patients’ sputum, urine, and other body fluids remains unclear. Although animal studies have reproduced the expression of some circRNAs in humans, the screening process for these circRNAs involves a small size of patients; thus, larger patient cohort studies are warranted. Moreover, even most circRNAs are reported as biomarkers for qualitative diagnosis, the correlation between the circRNA levels and the degree of disease severity is not well analyzed, and whether circRNA expression is associated with lung function requires further exploration.

Third, interpreting the role of macrophages in lung injury repair and fibrosis is complicated. In the lung injury stage, macrophages display a pro-inflammatory phenotype aggravating injury chief by M1. However, during the lung repair process, the function of M2 is dominant; it contributes to lung fibroblast cell proliferation and differentiation, leading to the risk of pulmonary fibrosis. The critical division in the circRNA balance of macrophage polarization is still unknown. Moreover, three different populations of macrophages are existed, namely, airway, alveolar, and interstitial macrophages (Jiang and Zhu, 2016; Hesketh et al., 2017; Joshi et al., 2018). The effect of circRNAs on different populations of macrophages requires further research. In addition to macrophages, other inflammatory cells, such as neutrophils, are involved in the inflammatory process. Whether circRNAs are involved in regulating neutrophils in the process of lung injury needs to be further explored.

What’s more, antisense oligonucleotides are artificially synthesized specific nucleic acid sequences that specifically target ncRNAs, such as lncRNAs and circRNAs. They have been approved by the FDA for clinical application, making it possible for selectively targeted circRNAs to be used in diagnostic and therapeutic approaches (Chan et al., 2006; Das et al., 2021). Whether the antisense oligonucleotides can be used for lung injury treatment are still a long way off.

In summary, circRNA has great potential as a diagnostic, therapeutic and prognostic target in lung injury diseases, fully elucidating the underlying mechanism of circRNAs in lung injury may radically improve the treatment. Continuous development of biotechnology and further exploration of circRNAs would greatly benefit patients with ALI.
